# Benign‐malignant classification of pulmonary nodules by low‐dose spiral computerized tomography and clinical data with machine learning in opportunistic screening

**DOI:** 10.1002/cam4.5886

**Published:** 2023-05-29

**Authors:** Yansong Zheng, Jing Dong, Xue Yang, Ping Shuai, Yongli Li, Hailin Li, Shengyong Dong, Yan Gong, Miao Liu, Qiang Zeng

**Affiliations:** ^1^ Department of Health Medicine Second Medical Center & National Clinical Research Center for Geriatric Diseases Chinese People's Liberation Army General Hospital Beijing China; ^2^ Research of Medical Big Data Center & National Engineering Laboratory for Medical Big Data Application Technology Chinese PLA General Hospital Beijing China; ^3^ Health Management Center Sichuan Provincial People's Hospital University of Electronic Science and Technology of China Chengdu China; ^4^ Department of Health Management/ Henan Provincial People's Hospital of Zhengzhou University Henan Key Laboratory of Chronic Disease Management Zhengzhou China; ^5^ Beijing Advanced Innovation Center for Big Data‐Based Precision Medicine School of Medicine and Engineering Beihang University Beijing China; ^6^ CAS Key Laboratory of Molecular Imaging Institute of Automation Beijing China; ^7^ Graduate School Chinese PLA general hospital Beijing China

**Keywords:** cancer screening, health examination, low‐dose computed tomography, lung cancer, opportunistic screening, pulmonary nodules

## Abstract

**Background:**

Many people were found with pulmonary nodules during physical examinations. It is of great practical significance to discriminate benign and malignant nodules by using data mining technology.

**Methods:**

The subjects' demographic data, baseline examination results, and annual follow‐up low‐dose spiral computerized tomography (LDCT) results were recorded. The findings from annual physical examinations of positive nodules, including highly suspicious nodules and clinically tentative benign nodules, was analyzed. The extreme gradient boosting (XGBoost) model was constructed and the Grid Search CV method was used to select the super parameters. External unit data were used as an external validation set to evaluate the generalization performance of the model.

**Results:**

A total of 135,503 physical examinees were enrolled. Baseline testing found that 27,636 (20.40%) participants had clinically tentative benign nodules and 611 (0.45%) participants had highly suspicious nodules. The proportion of highly suspicious nodules in participants with negative baseline was about 0.12%–0.46%, which was lower than the baseline level except the follow‐up of >5 years. In the 27,636 participants with clinically tentative benign nodules, only in the first year of LDCT re‐examination was the proportion of highly suspicious nodules (1.40%) significantly greater than that of baseline screening (0.45%) (*p* < 0.001), and the proportion of highly suspicious nodules was not different between the baseline screening and other follow‐up years (*p* > 0.05). Furthermore, 322 cases with benign nodules and 196 patients with malignant nodules confirmed by surgery and pathology were compared. A model and the top 15 most important clinical variables were determined by XGBoost algorithm. The area under the curve (AUC) of the model was 0.76 [95% CI: 0.67–0.84], and the accuracy was 0.75. The sensitivity and specificity of the model under this threshold were 0.78 and 0.73, respectively. In the validation of model using external data, the AUC was 0.87 and the accuracy was 0.80. The sensitivity and specificity were 0.83 and 0.77, respectively.

**Conclusions:**

It is important that pulmonary nodules could be more accurately identified at the first LDCT examination. A model with 15 variables which are routinely measured in the clinic could be helpful to distinguish benign and malignant nodules. It could help the radiological team issue a more accurate report; and it may guide the clinical team regarding LDCT follow‐up.

## 1 INTRODUCTION

Lung cancer continues to be the cancer with the highest incidence and mortality rate worldwide, and has remained so for almost 40 years.^1^, ^2^, ^3^ Early detection of lung cancer mainly depends on the screening of low‐dose spiral computerized tomography (LDCT) in the lung.^4^ The proportion of pulmonary nodules found in LDCT screening has been shown to be as high as 30%, most of which are subsequently confirmed as benign. Despite the benign status, many patients still receive invasive examination and treatment such as surgery and puncture.^5^ A study by Gopal and co‐workers^6^ found that nine cases of early lung cancer were detected per 1000 people screened by LDCT, but among these, 235 false‐positive nodules were also detected. In some studies, it was believed that after learning that the pulmonary nodules were positive, rates of LDCT re‐examination increased. These excessive rates of LDCT testing are responsible for an increased economic burden on the healthcare system,^7^, ^8^ and furthermore expose patients to high doses of radiation for medically unnecessary reasons.^9^, ^10^ It has also been shown that LDCT re‐examination causes undue stress and emotional effects on patients.^11^

Recent medical reports found that lung cancer rates were increasing among youth and nonsmokers in China.^12^, ^13^ Given that rates are increasing and the practical and social significance of early lung cancer detection, it is obviously unwise to reduce the scope of LDCT screening and focus only on high‐risk groups.^14^ The more feasible method is to continuously expand the scope of screening^15^ while simultaneously improving the accuracy and predictability of screening methods. On the one hand, instrumentation performance can be enhanced to obtain clearer images, and technical personnel can be more rigorously trained in image analysis.^16^, ^17^, ^18^, ^19^, ^20^ On the other hand, with modern computational power, big data and artificial intelligence can be utilized to analyze clinical data more effectively. This latter approach shows the most potential to improve overall lung cancer screening quality and efficiency, and remains an important new direction of development.^21^, ^22^

The screening of lung cancer by physical examination continues to be opportunistic in its application.^23^ In China, opportunistic screening plays a vital role in the early detection of lung cancer.^24^ At present, there are many patients with pulmonary nodules found in routine physical examination, most of whom undergo regular review by LDCT every 3–12 months.^25^, ^26^ From the perspective of avoiding missed diagnosis, this strategy seems scientific and reasonable. However, for most people with benign nodules, the clinical and biological significance of this high‐frequency re‐examination has always been a concern among physicians and scientists. The present study used data mining technology to analyze clinical variables captured in physical examination of patients with positive pulmonary nodules, to determine whether computational methods can assist in the discrimination between benign and malignant nodules. On the one hand, it can help the radiological team issue a more accurate report; on the other, it can better guide the clinical team regarding LDCT follow‐up, thereby avoiding the unnecessary and undue risks and costs associated with this follow‐up.

## 2 METHODS

### 2.1 Participants

**FIGURE 1:**
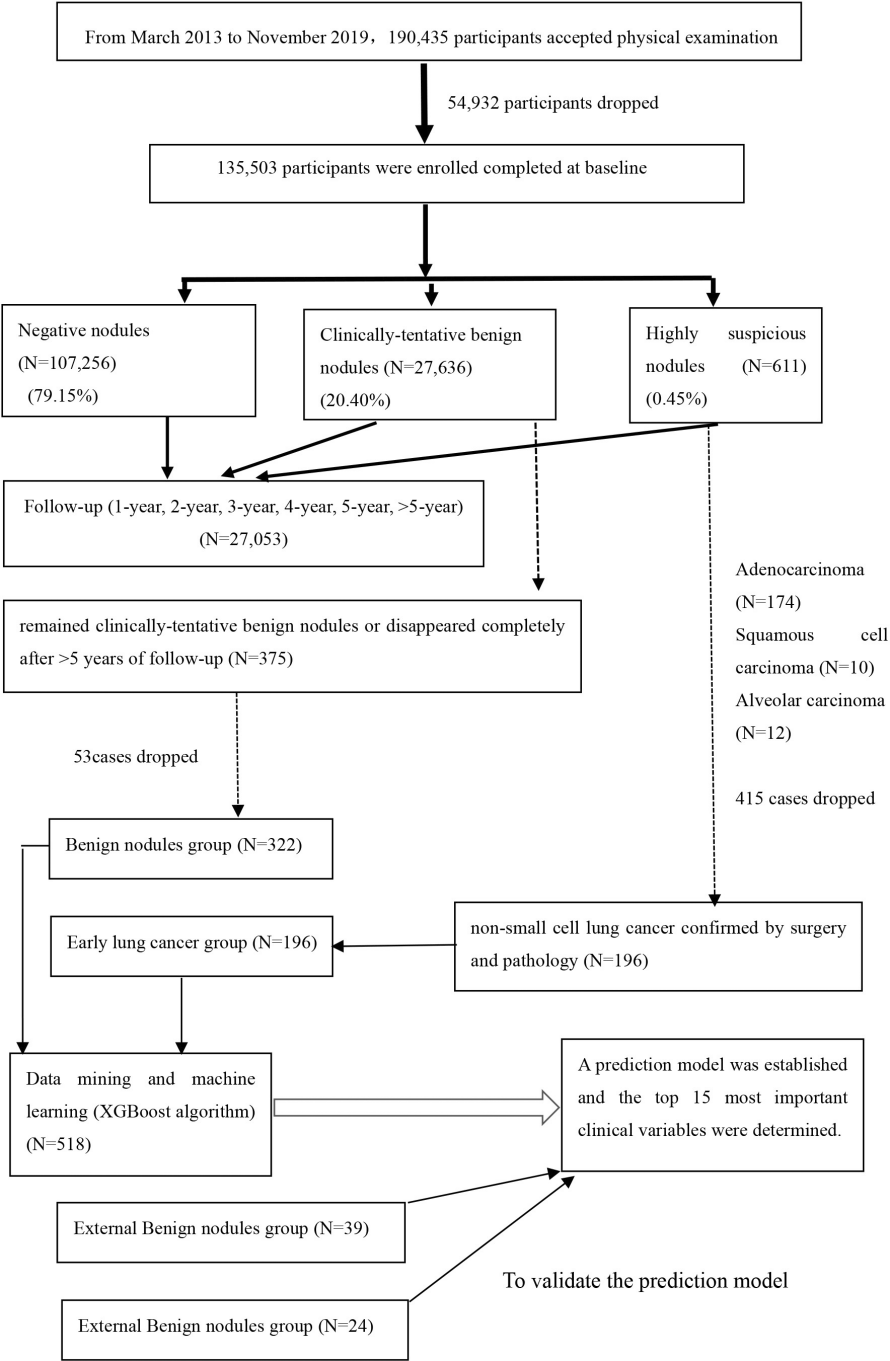
Flow diagram of enrolled participants.

Inclusion criterion: All subjects who underwent both physical examination lung LDCT screening in the Institute of Health Management of the General Hospital of the Chinese People's Liberation Army (PLA) from April 2013 to December 2019. Exclusion criteria: Patients with lung masses of >3 cm local lesion diameter; patients with >10 pulmonary nodules^27^; patients with severe liver/kidney dysfunction or cardiovascular disease; patients who have previously been diagnosed with lung cancer; patients who have been treated for space‐occupying lesions of the lung, have tumors in other organs, or are suspected of cancer metastasis. Participants included in the validation model were from Henan Provincial People's Medical Health Examination Center and Sichuan Provincial People's Hospital with similar inclusion and exclusion criteria as mentioned above (Figure 1). The study protocol was approved (S2021‐427‐01) by the Ethics Committee of Chinese People's Liberation Army General Hospital and complied with the principles of the Declaration of Helsinki and its contemporary amendments. The subjects were informed that their physical examination data may be used scientifically in a deidentified manner, and informed consent was obtained.

### 2.2 Data collection

The questionnaire was utilized to record in detail the subject's age (calculated according to the date of birth on the ID card), smoking and alcohol use, personal and family medical history, and environmental exposure. The personal medical history included any prior diagnosis of chronic obstructive pulmonary disease (COPD), tuberculosis and/or asthma, and whether the individual has a history of environmental or high‐risk occupational exposure (such as exposure to asbestos and radioactive toxic gas); the family history included prior diagnoses of lung cancer and/or other malignant tumors in immediate relatives. Smoking status definitions included the following: smoking ≥10 cigarettes a day for more than 1 year and those who had quit smoking within the past 5 years were regarded as smokers; those who had quit smoking >5 years were regarded as nonsmokers. Smoking amount = number of cigarettes per day × years of smoking. Alcohol use definitions included the following: no use or a small amount of alcohol use per day (≤25 g/day for males, ≤15 g/day for females), and habitual users (≥25 g/day for males, ≥15 g/day for females). Environmental exposures included living with a habitual smoker (i.e., continuous exposure to secondhand smoke) and high‐risk occupational exposure (e.g., asbestos, coal mine, radioactive toxic gas).

Participants wore uniform and loose clothes for physical examination under fasting state. Body weight, height, and body fat percentage were measured by using a bioelectrical impedance analyzer (InBody 720 analyzer, InBody Co. Ltd), and body mass index (BMI = body weight [kg]/body height [m^2^]) and skeletal muscle mass index (SMI = muscle mass/body weight × 100%) were calculated. After 10 min of rest, systolic and diastolic blood pressures were measured. Deep tissue ultrasound was used to determine the presence of fatty liver and thyroid nodules.

### 2.3 Determination of blood composition

On the same day of LDCT, venous blood was collected under the fasting condition. According to the quality control and testing standards of the Clinical Laboratory Department of the PLA General Hospital,^28^, ^29^ the following indicators were tested: total cholesterol, triglyceride (TG), high‐density lipoprotein cholesterol (HDL‐C), low‐density lipoprotein cholesterol, fasting blood glucose, blood uric acid (UA), hemoglobin A1c, serum creatinine (Cr) and blood urea nitrogen (Bun); squamous cell carcinoma associated antigen (SCC), alpha fetoprotein, carcinoembryonic antigen (CEA), neuron‐specific enolase, carbohydrate antigen 19‐9 (CA19‐9), cytokeratin, tumor antigen 12‐5, tumor antigen 15‐3, thyroglobulin antibody, thyroid peroxidase antibody, TSH, T3, T4, fT3, fT4, creatine kinase (Ck), Hcy (homocysteine), mean platelet volume, basophils, eosinophils, hemoglobin, platelet count, leukocyte count, reticulocyte percentage, monocytes, lymphocytes, neutrophils, erythrocyte volume distribution, erythrocyte count, hematocrit, mean erythrocyte volume, mean erythrocyte hemoglobin, mean erythrocyte hemoglobin concentration; aspartate aminotransferase (AST), alanine aminotransferase (ALT), direct bilirubin (DB), total bilirubin (TB), total protein (TP), alkaline phosphatase (ALP), γ‐glutamyltransferase (γ‐GGT), serum total bile acid, and albumin (ALB).

### 2.4 Determination of *Helicobacter pylori* infection

A ^13^C‐urea breath test (Helikit, AltaChem Pharma Ltd) was used to detect Hp infection of the gastric mucosa. None of the patients had taken antibiotics during the past month. Participants fasted for 4 h before the test. The obtained samples were analyzed by gas chromatography–isotope ratio mass spectrometry.^30^ The results were determined as positive or negative based on a device algorithm. The participants with a delta over baseline (the ^13^CO_2_/^12^CO_2_ ratio) ≥4 were considered positive (i.e., confirmed gastric Hp infection).

### 2.5 LDCT scanning

A tube voltage of 100–140 kVp was used according to the subject's weight. A tube current of <60 mA was used. The total radiation exposure dose was ≤1 mSv. The scanning area was from the apex of the lung to the costophrenic angle to include the entire lung. After scanning, raw data were used for thin slice reconstruction. The reconstructed slice thickness was 0.625–1.25 mm. Scanning range was from lung tip to costophrenic angle (including the whole lung). Scanning sampling time was ≤10 s, the respiratory phase was defined as the end of deep inhalation, and the CT scanning detector spanned ≥16 rows with no contrast agent required. Soft tissue density or lung algorithm was recommended for thin‐layer reconstruction. For the detection of pulmonary nodules, the maximum density projection was used for three‐dimensional reconstruction of thin‐layer images.^26^

### 2.6 Analysis of LDCT scans

Nodule‐positive scans were characterized as being positive for focal, quasi‐circular, dense solid, or sub‐solid (partially solid) ground‐glass lung shadows with 5 mm ≤ nodule diameter ≤ 30 mm. These could be isolated or multiple (≤10), without atelectasis, hilar lymphadenopathy and pleural effusion, as previously described.^31^ Highly suspicious nodules were designated according to the following criteria: (1) the diameter of pure ground‐glass nodules was ≥10 mm; or the presence of nodules whose diameter increased by ≥2 mm as compared with the baseline diameter of ≤15 mm; or those nodules whose baseline diameter was >15 mm and increased by more than 15% as compared with the baseline^32^; (2) the density of pure ground‐glass nodules increased or there were solid components in them; or the solid components of sub‐solid ground‐glass nodules with uneven density exceeded 50%^33^; (3) thickening of tracheal and bronchial walls, lumen stenosis, or intraluminal nodules; (4) the presence of angiogenesis consistent with the law of malignant pulmonary nodules; and (5) the presence of lobulation, burr, and/or pleural depression. All other nodules were designated as clinically tentative benign nodules. Patients with lung cancer confirmed by pathological results in the later stage were included in the early lung cancer group. Although there were no pathological results, patients with confirmed benign nodules or the disappearance of nodules were included in the benign nodule group. CT results were independently analyzed by two doctors.

### 2.7 Clinical follow‐up

Within the same subject, each LDCT re‐examination was regarded as one follow‐up. The first year of follow‐up was defined as any re‐examination with 365 days of initial LDCT scan; similarly, follow‐up of >1825 days was defined as >5 years. Patients with suspicious malignant nodules found at baseline examination were referred to the clinic for intervention. Subjects who underwent thoracic surgery in our hospital were tracked through ID number or through family members via telephone to understand the results of any later operation. If the clinically tentative benign nodules found at baseline remained as benign nodules in the LDCT re‐examination with an interval of >5 years, or the nodules disappeared in the subsequent follow‐up, then all pulmonary nodules found at baseline in those patients were defined as benign nodules.

### 2.8 XGBoost machine learning model

XGBoost (Extreme Gradient Boosting) is an integrated learning algorithm based on the gradient boosting decision tree. The objective function is Taylor‐expanded to the second order to make the gradient decline faster and more accurate, and the regularization term is introduced to control the complexity of the model and prevent over‐fitting. Automatically processing missing values greatly improves the efficiency of the algorithm. When splitting the decision tree, features are selected based on the information gain. The more times a feature is selected to split, the greater its average gain (i.e., it is proved to be an important variable).

### 2.9 Statistical analysis

Questionnaire data were encoded, quantified, and input into the computer, and statistical analysis was carried out using Stata 11.0 software. The Kolmogorov–Smirnov method was used to test for normality, the classification data were expressed by frequency and rate, and the independent sample *t*‐test and chi‐squared test were used to determine main effect differences between groups with *p* < 0.05 designated as statistically significant. All data were divided into training set and test set in a ratio of 8:2; the XGBoost machine learning model was constructed with python 3.8. The Grid Search cross‐validation method was used to select the super parameters. External unit data were used as an external validation set to evaluate the generalization performance of the model. To evaluate the prediction performance of the model, receiver operating characteristic curves (ROCs) were used to calculate the area under the curve (AUC), and the accuracy, sensitivity, and specificity were determined based on the threshold of 0.5.

## 3 RESULTS

### 3.1 Clinical features

A total of 135,503 people underwent physical examination and lung LDCT screening during the study period. The average subject age was 47.96 ± 9.93 years; there were 89,705 males (66.20%) and 45,798 females (33.80%). The baseline LDCT examination of 135,503 subjects showed that 107,256 persons (79.15%) had negative nodules. Clinically tentative benign nodules were found in 27,636 cases, accounting for 20.40%. There were 611 highly suspicious nodules, accounting for 0.45%.

### 3.2 LDCT follow‐up screening

A total of 27,053 LDCT follow‐ups were completed in 135,503 participants (Table 1). The results showed that the proportion of highly suspicious nodules in participants with negative baseline was about 0.12%–0.46%, which was lower than the baseline level except the follow‐up of >5 years. During follow‐up, 16.5%–27.5% of the participants with clinically tentative benign nodules at baseline turned negative, 71.53%–81.95% remained benign, and the proportion of highly suspicious nodules was higher at follow‐up than that of the baseline screening (0.70% vs. 0.45%) (χ^2^ = 8.09, *p* = 0.004). However, only in the first year of LDCT re‐examination was the proportion of highly suspicious nodules (1.40%) significantly greater than that of baseline screening (0.45%) (*p* < 0.001), and the proportion of highly suspicious nodules was not different between the baseline screening and other follow‐up years (*p* > 0.05). This suggested that if he or she was diagnosis as clinically tentative benign nodules in the opportunistic screening, it was important to review LDCT within 1 year, and the follow‐up value of more than 1 year is not greater than that of random opportunistic screening.

**TABLE 1:** Follow up results of low‐dose computed tomography opportunistic screening.

	Follow‐up status	Baseline			Total
		Nodule negative	Tentative benign nodules	Highly suspicious nodules	
1 year (*N* = 3871)	Nodule negative (%)	2561 (88.86%)	155 (16.65%)	16 (27.59%)	2732 (70.58%)
	Tentative benign nodules (%)	315 (10.93%)	763 (81.95%)	13 (22.41%)	1091 (28.18%)
	Highly suspicious nodules (%)	6 (0.21%)^a^	13 (1.40%)^a^	29 (50.00%)	48 (1.24%)
	Total	2882 (100.00%)	931 (100.00%)	58 (100.00%)	3871 (100.00%)
2 years (*N* = 8845)	Nodule negative (%)	5775 (85.72%)	448 (21.72%)	12 (26.67%)	6235 (70.49%)
	Tentative benign nodules (%)	949 (14.09%)	1604 (77.75%)	17 (37.78%)	2570 (29.06%)
	Highly suspicious nodules (%)	13 (0.19%)^a^	11 (0.53%)	16 (35.56%)	40 (0.45%)
	Total	6737 (100.00%)	2063 (100.00%)	45 (100.00%)	8845 (100.00%)
3 years (*N* = 6661)	Nodule negative (%)	4298 (83.38%)	356 (24.27%)	8 (20.51%)	4662 (69.99%)
	Tentative benign nodules (%)	845 (16.39%)	1105 (75.32%)	22 (56.41%)	1972 (29.61%)
	Highly suspicious nodules (%)	12 (0.23%)^a^	6 (0.41%)	9 (23.08%)†	27 (0.41%)
	Total	5155 (100.00%)	1467 (100.00%)	39 (100.00%)	6661 (100.00%)
4 years (*N* = 4021)	Nodule negative (%)	2485 (80.34%)	226 (25.00%)	8 (33.33%)	2719 (67.62%)
	Tentative benign nodules (%)	600 (19.40%)	670 (74.12%)	11 (45.83%)	1281 (31.86%)
	Highly suspicious nodules (%)	8 (0.26%)^a^	8 (0.88%)	5 (20.83%)†	21 (0.52%)
	Total	3093 (100.00%)	904 (100.00%)	24 (100.00%)	4021 (100.00%)
5 years (*N* = 2181)	Nodule negative (%)	1221 (75.42%)	151 (27.55%)	6 (42.86%)	1378 (63.18%)
	Tentative benign nodules (%)	396 (24.46%)	392 (71.53%)	4 (28.57%)	792 (36.31%)
	Highly suspicious nodules (%)	2 (0.12%)^a^	5 (0.91%)	4 (28.57%)	11 (0.50%)
	Total	1619 (100.00%)	548 (100.00%)	14 (100.00%)	2181 (100.00%)
>5 years (*N* = 1474)	Nodule negative (%)	782 (71.74%)	95 (25.27%)	2 (25.00%)	879 (59.63%)
	Tentative benign nodules (%)	303 (27.80%)	280 (74.47%)	5 (62.50%)	588 (39.89%)
	Highly suspicious nodules (%)	5 (0.46%)	1 (0.27%)	1 (12.50%)^b^	7 (0.47%)
	Total	1090 (100.00%)	376 (100.00%)	8 (100.00%)	1474 (100.00%)
Total (*N* = 27,053)	Nodule negative (%)	17,122 (83.21%)	1431 (22.75%)	52 (27.66%)	18,605 (68.77%)
	Tentative benign nodules (%)	3408 (16.56%)	4814 (76.55%)	72 (38.30%)	8294 (30.66%)
	Highly suspicious nodules (%)	46 (0.22%)	44 (0.70%)	64 (34.04%)	154 (0.57%)
	Total	20,576 (100.00%)	6289 (100.00%)	188 (100.00%)	27,053 (100.00%)

^a^ Compared with baseline examination, 611 participants had highly suspicious nodules and others did not show highly suspicious nodules, *p* < 0.05.

^b^ Compared with 1‐year follow‐up, *p* < 0.05.

In total, the proportion of highly suspicious nodules in subjects who completed follow‐up was higher than that of baseline screening (0.57% vs. 0.45%) (χ^2^ = 6.74, *p* = 0.009); it was only in the first year of LDCT re‐examination when the proportion of highly suspicious nodules was significantly higher than at baseline (1.24% vs. 0.45%) (χ^2^ = 6.74, *p* = 0.009). Furthermore, the proportion of highly suspicious nodules was not different between the baseline screening and other follow‐up years. The above results suggest that if we could accurately find those nodules, and distinguish benign and malignant nodules, we could avoid many unnecessary LDCT examinations.

### 3.3 Comparison of baseline physical examination results between benign nodule group and lung cancer patients

Among the 611 patients with suspected malignant nodules found in the baseline examination, 196 cases were confirmed as lung cancer by surgery and pathology, predominantly non‐small cell lung cancer. There were 174 cases of adenocarcinoma, 10 cases of squamous cell carcinoma, and 12 cases of alveolar carcinoma. Among 27,636 cases of clinically tentative benign nodules found at baseline, 375 cases remained benign or disappeared completely after >5 years of follow‐up. The 53 cases with incomplete baseline data were excluded, and 322 cases were included in the benign nodule group (Figure 1). Tables 2 and 3 show comparisons of baseline physical examination results between the lung cancer and benign nodule groups.

**TABLE 2:** Comparison of baseline physical examination classification results between benign nodule group and early lung cancer group.

Category	Early lung cancer group (*n* = 196)	Benign nodule group (*n* = 322)	Statistics
Gender			χ^2^ = 18.68, *p* < 0.01
Female	83 (42.35)	78 (24.22)	
Male	113 (57.65)	244 (75.78)	
Personal history of COPD, asthma, tuberculosis			χ^2^ = 17.18, *p* < 0.01
Negative	186 (94.90)	265 (82.30)	
Positive	10 (5.10)	57 (17.70)	
Family history of illness			
Lung cancer	15 (7.65)	28 (8.70)	χ^2^ = 0.17, *p* = 0.68
Malignant tumor	17 (8.67)	33 (10.25)	χ^2^ = 0.35, *p* = 0.56
COPD, asthma, family history of tuberculosis	9 (4.59)	55 (17.08)	χ^2^ = 17.55, *p* < 0.01
Smoking status			χ^2^ = 22.85, *p* < 0.01
Never smoking	95 (46.94)	168 (52.17)	
Quit smoking	5 (2.55)	42 (13.04)	
Smoking	99 (50.51)	112 (34.78)	
Smoking index	318.95348.52	248.56315.95	*z* = 2.22, *p* = 0.03
Drinking			χ^2^ = 8.83, *p* < 0.01
Never or small amount of alcohol	94 (47.96)	112 (34.78)	
Yes	102 (52.04)	210 (65.22)	
Environmental exposure			
Secondhand smoke	186 (94.9)	238 (73.91)	χ^2^ = 36.12, *p* < 0.01
High‐risk occupational environment	2 (1.02)	35 (10.87)	χ^2^ = 17.82, *p* < 0.01
Tumor markers			χ^2^ = 68.52, *p* < 0.01
Negative	68 (34.69)	231 (71.74)	
Positive (≥1 positive marker)	128 (65.31)	91 (28.26)	
Abnormal blood sugar			χ^2^ = 2.66, p = 0.10
No	145 (73.98)	258 (80.12)	
Yes	51 (26.02)	64 (19.88)	
High blood pressure			χ^2^ = 3.38, *p* = 0.07
No	112 (57.14)	210 (65.22)	
Yes	84 (42.86)	112 (34.78)	
Dyslipidemia			χ^2^ = 0.13, *p* = 0.72
No	70 (35.71)	120 (37.27)	
Yes	126 (64.29)	202 (62.73)	
Uric acid abnormalities			χ^2^ = 11.22, *p* < 0.01
No	188 (95.92)	280 (86.96)	
Yes	8 (4.08)	42 (13.04)	
Liver enzyme abnormalities			χ^2^ = 0.71, *p* = 0.40
No	149 (76.02)	234 (72.67)	
Yes	47 (23.98)	88 (27.33)	
Thyroid function			χ^2^ = 0.18, p = 0.67
Normal	141 (71.94)	226 (70.19)	
Abnormal	55 (28.06)	96 (29.81)	
Hypertension			χ^2^ = 0.35, *p* = 0.55
No	124 (63.27)	212 (65.84)	
Yes	72 (36.73)	110 (34.16)	
Diabetes			χ^2^ = 0.78, *p* = 0.38
No	163 (83.16)	277 (86.03)	
Yes	33 (16.84)	45 (13.98)	
Fatty liver			χ^2^ = 1.35, *p* = 0.25
No	85 (43.37)	123 (38.20)	
Yes	111 (56.63)	199 (61.80)	
Thyroid nodules			χ^2^ = 4.33, *p* = 0.04
No	85 (43.37)	170 (52.80)	
Yes	111 (56.63)	152 (47.20)	
*Helicobacter pylori* infection			χ^2^ = 0.16, *p* = 0.69
No	125 (63.78)	211 (65.53)	
Yes	71 (36.22)	111 (34.47)	

**TABLE 3:** Comparison of baseline physical examination results between benign nodules and early lung cancer.

Category	Early lung cancer group (*n* = 196)	Benign nodule group (*n* = 322)	Statistics
Age (year)	56.87 ± 9.62	51.62 ± 8.12	*z* = 6.55, *p* < 0.01
Fasting blood glucose (mmol/L)	5.87 ± 1.78	5.58 ± 1.24	*z* = 1.27, *p* = 0.20
Systolic blood pressure (mmHg)	126.08 ± 17.34	121.65 ± 15.35	*z* = 2.91, *p* < 0.01
Diastolic blood pressure (mmHg)	80.15 ± 10.40	78.27 ± 9.45	*z* = 2.34, *p* = 0.02
HbA1c (%)	6.04 ± 1.06	5.68 ± 0.73	*z* = 5.35, *p* < 0.01
TC (mmol/L)	4.73 ± 0.90	4.76 ± 0.89	*z* = 0.20, *p* = 0.84
TG (mmol/L)	1.63 ± 0.99	1.87 ± 2.26	*z* = 1.86, *p* = 0.06
HDL‐C (mmol/L)	1.20 ± 0.33	1.21 ± 0.33	*z* = 0.58, *p* = 0.56
LDL‐C (mmol/L)	3.07 ± 0.81	2.99 ± 0.77	*z* = 0.94, *p* = 0.35
UA (μmol/L)	316.10 ± 77.99	354.84 ± 79.26	*z* = 5.10, *p* < 0.01
AST (U/L)	19.98 ± 7.54	21.98 ± 13.43	*z* = 2.21, *p* = 0.03
ALT (U/L)	22.36 ± 14.20	27.32 ± 26.84	*z* = 2.75, *p* = 0.01
ALP (U/L)	73.76 ± 61.11	65.51 ± 18.06	*z* = 1.71, *p* = 0.09
γ‐GGT (U/L)	33.97 ± 31.63	40.14 ± 45.48	*z* = 2.01, *p* = 0.04
TSH (mU/L)	2.54 ± 1.90	2.50 ± 1.91	*z* = 0.50, *p* = 0.62
TT3 (nmol/L)	1.68 ± 0.27	1.71 ± 0.28	*z* = 1.33, *p* = 0.19
TT4 (nmol/L)	100.32 ± 18.37	100.16 ± 18.44	*z* = 0.34, *p* = 0.73
FT3 (pmol/L)	4.68 ± 0.56	4.96 ± 0.69	*z* = 4.39, *p* < 0.01
FT4 (pmol/L)	16.11 ± 2.09	16.37 ± 2.39	*z* = 0.86, *p* = 0.39
TG‐Ab (IU/mL)	52.99 ± 267.25	58.18 ± 251.89	*z* = 1.13, *p* = 0.26
TPO‐Ab (IU/mL)	22.86 ± 69.28	27.89 ± 78.41	*z* = 0.03, *p* = 0.98
Alpha‐fetoprotein (AFP) (μg/L)	3.56 ± 7.56	3.07 ± 1.50	*z* = 0.22, *p* = 0.83
CEA (μg/L)	48.88 ± 508.48	2.00 ± 1.27	*z* = 1.85, *p* = 0.06
NSE (ng/mL)	13.35 ± 19.34	11.26 ± 2.99	*z* = 1.37, *p* = 0.17
CA19‐9 (U/mL)	13.55 ± 15.18	11.04 ± 9.11	*z* = 2.17, *p* = 0.03
Cyfra 21‐1 (ng/mL)	2.88 ± 2.41	2.16 ± 0.89	*z* = 3.48, *p* < 0.01
CA12‐5 (U/mL)	42.63 ± 392.67	10.18 ± 7.05	*z* = 2.69, *p* = 0.01
CA15‐3 (U/mL)	15.70 ± 29.80	10.89 ± 5.53	*z* = 1.54, *p* = 0.12
SCC (ng/mL)	0.97 ± 0.72	0.93 ± 0.36	*z* = 1.79, *p* = 0.07
Blood routine			
Mean platelet volume determination (fL)	10.54 ± 0.83	10.66 ± 0.96	*z* = 1.05, *p* = 0.29
Basophils (100%)	0.43 ± 0.27	0.42 ± 0.29	*z* = 0.68, *p* = 0.49
Eosinophils (100%)	8.60 ± 12.84	13.85 ± 15.79	*z* = 6.52, *p* < 0.01
Hemoglobin determination (g/L)	141.48 ± 16.26	149.40 ± 13.57	*z* = 5.53, *p* < 0.01
Platelet count (10^9^/L)	225.76 ± 58.58	210.54 ± 50.24	*z* = 3.16, *p* < 0.01
White blood cell count (10^9^/L)	6.25 ± 1.89	5.98 ± 1.46	*z* = 1.32, *p* = 0.19
Percentage of reticulocytes (100%)	1.41 ± 0.45	1.41 ± 0.44	*z* = 0.16, *p* = 0.87
Monocyte (100%)	6.06 ± 1.69	6.23 ± 1.73	*z* = 1.03, *p* = 0.30
Lymphocytes (100%)	32.43 ± 8.21	33.85 ± 7.87	*z* = 1.93, *p* = 0.05
Neutrophils (100%)	58.53 ± 8.61	56.74 ± 8.55	*z* = 2.32, *p* = 0.02
Red blood cell count (10^12^/L)	4.66 ± 0.50	4.87 ± 0.43	*z* = 4.86, *p* < 0.01
Hematocrit determination (100%)	42.04 ± 4.37	44.28 ± 3.68	*z* = 5.95, *p* < 0.01
Mean erythrocyte volume (fL)	90.26 ± 4.98	90.90 ± 4.39	*z* = 2.12, *p* = 0.03
Mean erythrocyte hemoglobin (pg)	30.37 ± 1.84	30.69 ± 1.59	*z* = 2.24, *p* = 0.03
Mean erythrocyte hemoglobin concentration (g/L)	336.61 ± 13.73	337.69 ± 10.83	*z* = 0.66, *p* = 0.51
HCY (μmol/L)	13.99 ± 7.63	14.54 ± 7.35	*z* = 1.36, *p* = 0.17
CK (U/L)	96.33 ± 113.85	97.49 ± 82.08	*z* = 1.16, *p* = 0.25
ALB (g/L)	43.97 ± 3.34	45.22 ± 3.11	*z* = 4.09, *p* < 0.01
TP (g/L)	70.02 ± 5.36	71.11 ± 5.32	*z* = 2.35, *p* = 0.02
TB (μmol/L)	10.58 ± 4.96	11.38 ± 4.60	*z* = 2.40, *p* = 0.02
DB (μmol/L)	3.73 ± 1.47	3.81 ± 1.92	*z* = 0.73, *p* = 0.47
Total bile acid	4.62 ± 4.65	3.80 ± 3.92	*z* = 2.98, *p* < 0.01
Cr (μmol/L)	67.25 ± 14.93	71.70 ± 14.20	*z* = 3.73, *p* < 0.01
Bun (mmol/L)	5.34 ± 1.37	5.29 ± 1.29	*z* = 0.81, *p* = 0.42
Body fat percentage (BFP)	27.68 ± 6.88	27.47 ± 7.94	*z* = 0.95, *p* = 0.34
BMI	25.02 ± 3.21	25.14 ± 2.99	*z* = 0.78, *p* = 0.44
SMI (100%)	66.97 ± 6.57	67.84 ± 7.77	*z* = 1.82, *p* = 0.07

Abbreviations: γ‐GGT, γ‐glutamyltransferase; AFP, alpha‐fetoprotein; ALB, albumin; ALP, alkaline phosphatase; ALT, alanine aminotransferase; AST, aspartate aminotransferase; BMI, body mass index; Bun, blood urea nitrogen; CA12‐5, tumor antigen 12‐5; CA15‐3, tumor antigen 15‐3; CA19‐9, carbohydrate antigen 19‐9; CEA, carcinoembryonic antigen; Ck, creatine kinase; Cr, serum creatinine; Cyfra 21‐1, cytokeratin; DB, direct bilirubin; FBG, fasting blood glucose; HbA1c, hemoglobin A1c; Hcy, homocysteine; HDL‐C, high‐density lipoprotein cholesterol; LDL‐C, low‐density lipoprotein cholesterol; NSE, neuron‐specific enolase; SCC, squamous cell carcinoma–associated antigen; SMI:skeletal muscle mass index; TB, total bilirubin; TC, total cholesterol; TG, triglyceride; TG‐Ab, thyroglobulin antibody; TP, total protein; TPO‐Ab, thyroid peroxidase antibody; UA, blood uric acid.

### 3.4 Data mining and machine learning

**FIGURE 2:**
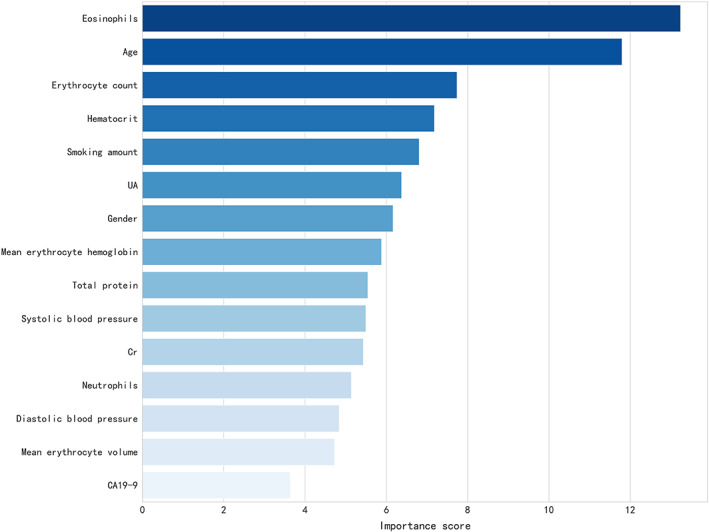
The top 15 clinical variables filtered by XGBoost algorithm.

**FIGURE 3:**
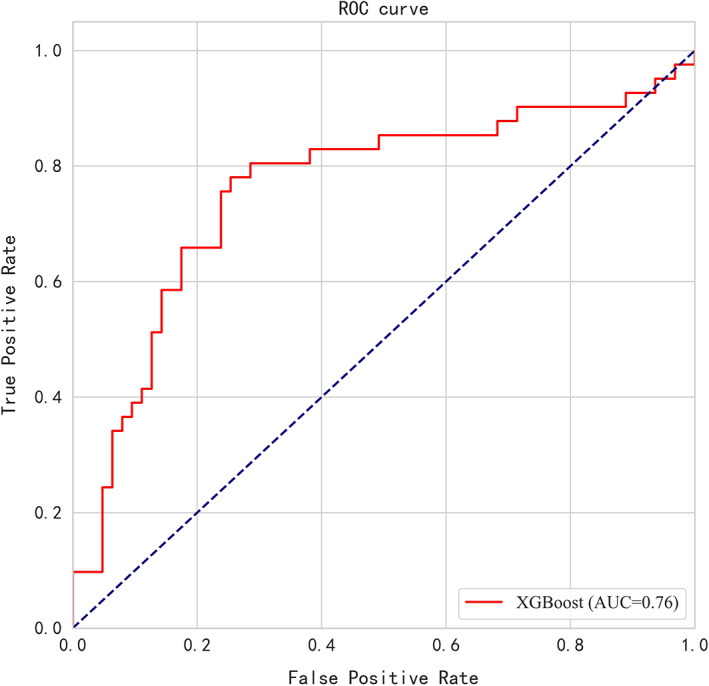
Receiver operating characteristic curve (ROC) of test set.

Since the physical examination results may be clinically related to pulmonary nodule status (i.e., benign vs. malignant), a computational approach was implemented using all the above variables as scalars and analyzed them with data mining technology. A prediction model was established and the top 15 most important clinical variables determined by XGBoost algorithm included eosinophil count, age, erythrocyte count, determination of hematocrit, smoking, UA, gender, average erythrocyte hemoglobin, total protein, systolic blood pressure, blood creatinine, neutrophils, diastolic blood pressure, average erythrocyte volume, and CA19‐9 (Figure 2). The model was verified in the test set. The AUC of the model was 0.76 [95% CI: 0.67–0.84], and the accuracy was 0.75. The sensitivity and specificity of the model were 0.78 and 0.73, respectively. The ROC is shown in Figure 3.

### 3.5 Validation of model using external data

**FIGURE 4:**
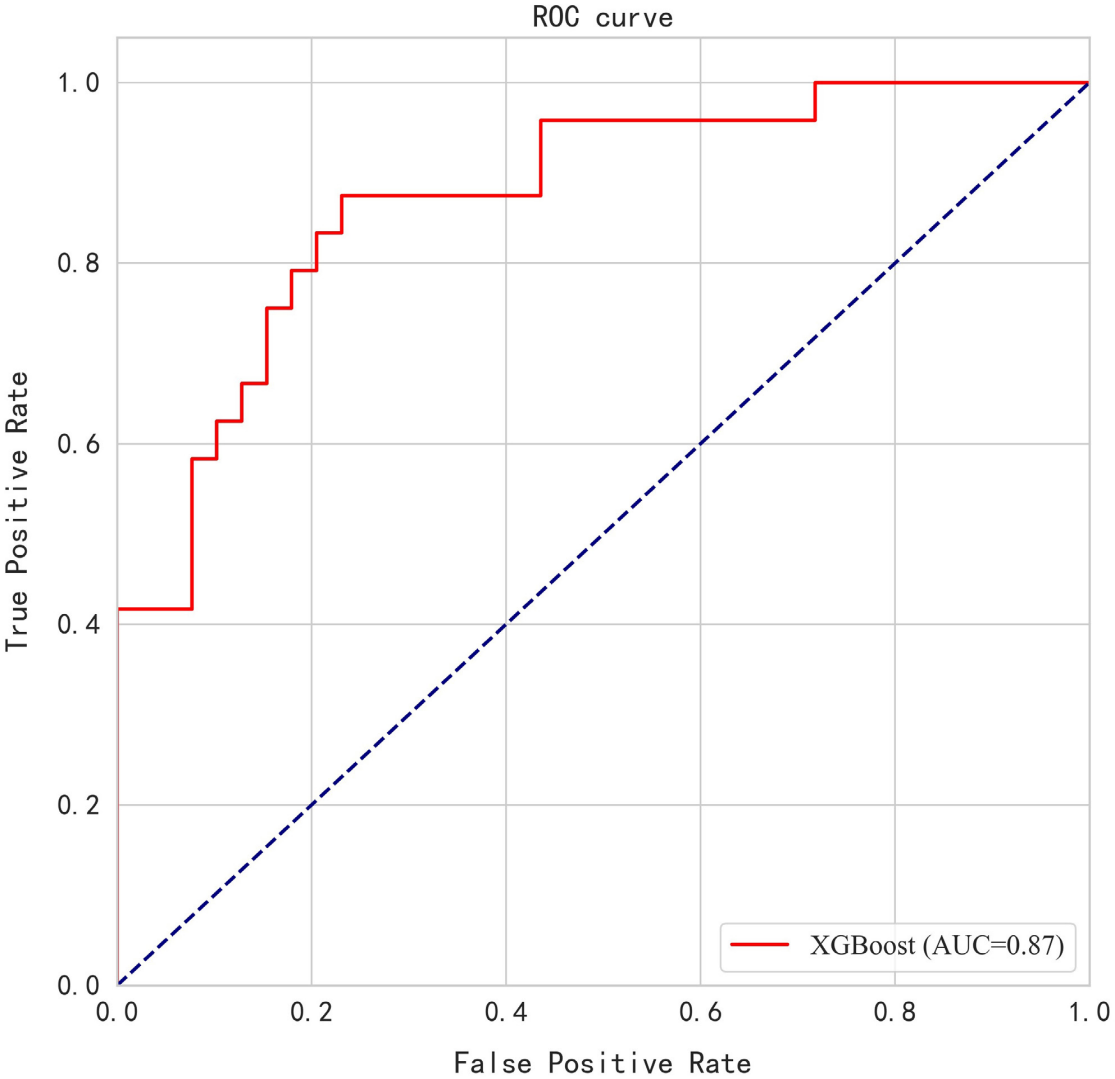
Receiver operating characteristic curve (ROC) for external data validation.

Data used in the validation model were from patients evaluated at Henan Provincial People's Medical Health Examination Center and Sichuan Provincial People's Hospital. The inclusion and exclusion criteria were the same as above. Among the 5146 patients screened in this validation data set, 24 cases were identified as early lung cancer, and the pathological results were adenocarcinoma. Benign nodules were found in 39 cases. The comparison of the 15 clinically relevant variables identified using the XGBoost algorithm in this validation cohort is shown in Table 4. In this validation cohort, the model AUC was 0.87, and the accuracy was 0.80. The sensitivity and specificity were 0.83 and 0.77, respectively. The ROC is shown in Figure 4.

**TABLE 4:** Comparison of 15 indicators of external verification data.

Category	Early lung cancer group (*n* = 24)	Benign nodule group (*n* = 39)	Statistics
Eosinophils	2.71 ± 2.29	2.78 ± 1.53	*z* = 1.063, *p* = 0.2880
Gender			χ^2^ = 4.2419, *p* = 0.058
Male	14	32	
Female	10	7	
Hematocrit (100%)	30.21 ± 3.74	40.18 ± 3.69	*z* = 5.386, *p* < 0.001
Age (years)	59.08 ± 13.63	50.64 ± 11.62	*z* = 2.421, *p* = 0.0155
Smoking amount	62.5 ± 120.91	86.67 ± 199.96	*z* = 0.038, *p* = 0.9697
Mean erythrocyte volume (fL)	93.54 ± 5.36	93.64 ± 6.34	*z* = 0.340, *p* = 0.7339
Diastolic blood pressure (mmHg)	75.25 ± 10.50	77.62 ± 11.88	*z* = 0.545, *p* = 0.5855
UA	331.42 ± 113.93	381.56 ± 80.68	*z* = 2.300, *p* = 0.0214
Red blood cell count (10^12^/L)	4.19 ± 0.37	4.94 ± 0.39	*z* = 5.605, *p* < 0.001
TP (g/L)	70.44 ± 3.44	74.46 ± 3.85	*z* = 3.921, *p* < 0.001
Cr (μmol/L)	64.21 ± 11.58	90.02 ± 86.79	*z* = 3.482, *p* = 0.0005
CA19‐9 (mmol/L)	12.33 ± 8.47	9.04 ± 6.36	*z* = 1.699, *p* = 0.0894
Neutrophils (100%)	56.26 ± 9.09	56.58 ± 6.21	*z* = 0.354, *p* = 0.7234
Systolic blood pressure (mmHg)	130.13 ± 22.42	125.67 ± 19.31	*z* = 0.807, *p* = 0.4196
Mean erythrocyte hemoglobin (pg)	30.93 ± 1.79	30.68 ± 2.22	*z* = 0.227, *p* = 0.8207

Abbreviations: CA19‐9, carbohydrate antigen 19‐9; Cr, serum creatinine; TP, total protein; UA, blood uric acid.

## 4 DISCUSSION

With respect to lung cancer screening, the value of opportunistic screening remains controversial.^31^, ^34^ Despite this controversy, LDCT screening has emerged as the most reliable and rigorous method of early lung disease detection among healthy people.^19^ A common chest CT typically delivers more than a hundred times the radiation dose of a routine frontal and lateral chest X‐ray (0.02–0.2 mSv, which is often quoted as 8–10 mSv). However, the total radiation exposure dose of LDCT in our study was ≤1 mSv, which is about 5–50 times of X‐ray.^35^

In the present study, baseline LDCT screening identified 107,256 persons (79.15%) with negative nodules, 27,636 persons (20.40%) with clinically tentative benign nodules, and 611 patients (0.45%) with highly suspicious nodules. It is notable that most nodules identified with LDCT screening in the baseline examination were determined to be clinically tentative benign nodules, which is consistent with previous reports.^4^ The proportion of highly suspicious nodules is significantly higher than the reported incidence of lung cancer in China (28.49/100,000).^36^, ^37^ This may be related to the fact that most of the subjects screened were middle‐aged and elderly people, with an average age of 47.96 ± 9.93 years. It is also important to note that many of these 611 highly suspicious nodules were determined not to be lung malignancies as confirmed by pathology.

In the population with negative nodules at baseline, the proportion of highly suspicious nodules was lower than the baseline level (*p* < 0.05), only reached 0.46% during >5 year follow‐up. This suggested that if no pulmonary nodules were found in the opportunistic screening, the probability of highly suspicious nodules within the next 5 years was not high, which was lower than the random opportunistic screening. In the population with clinically tentative benign nodules found in the baseline screening, it was determined that the proportion of highly suspicious nodules increased only within the first year of LDCT follow‐up, while the proportion of highly suspicious nodules was not different between other follow‐up years and the baseline screening. This suggests that in patients with pulmonary nodules for 2–5 years, or even more than 5 years, results of LDCT re‐examination were the same as that of the population receiving the first screening of LDCT. These findings are clinically significant given the psychological impact after finding nodules,^10^ the economic cost of re‐examination^36^ and radiation burden,^38^ because they suggest that multiple LDCT re‐examinations after 1 year cannot significantly increase the value of screening even if nodules are found with the initial screening. More importantly, the findings suggest that if malignant nodules can be more accurately identified at the first LDCT examination, the proportion of highly suspicious nodules found by the LDCT re‐examination may decline in the first year of follow‐up.

Based on this finding, we carried out further analysis on the population with pulmonary nodules by comparing patients with surgically confirmed early lung cancer with those whose nodules disappeared or remained benign after >5 years of follow‐up. In the baseline physical examination of these subjects, there were clear differences in age, gender distribution, smoking, environmental exposure, and many blood‐borne variables (Tables 3 and 4). However, it is very difficult to analyze whether these variables were affected by age and gender distribution, or whether they were really related to the status of the nodules. Therefore, we used machine learning technology and XGBoost algorithm to establish a prediction model of benign and malignant nodules using these variables.

The clinical variables included in this study were from the comprehensive physical examination of each subject. Statistically significant differences in many variables between patients with early lung cancer and those with benign nodules were initially identified with our analysis. However, after further analysis using XGBoost, it was found that the auxiliary prediction value of only 15 common clinical variables was significant. Moreover, these 15 variables were routinely measured in the clinic, and easy to obtain at most institutions. Therefore, our results also provide a good prerequisite for the universal application of this prediction method. Although the exact relationship between these variables and early lung cancer cannot remain unknown at the present time, our preliminary findings undoubtedly provide an alternative means of evaluating and stratifying lung cancer risk in patients with pulmonary nodules. This method could not only be useful for imaging experts, but may also provide clinicians with an additional tool to decide appropriate care for patients with nodules detected by LDCT. This evaluation model can be easily implanted into doctors' computers. By entering these 15 variables, it can be evaluated quickly. This method may help radiologists improve the accuracy of the diagnosis of pulmonary nodules. Compared with the standard of care, this method can help clinicians give patients clear suggestions, especially when those pulmonary nodules are ambiguous. Generally, the sensitivity and specificity of internal verification are higher than those of external verification. However, it was the opposite in our results. This may be due to different validation populations. Because subjects used in this study were from the population of patients who have undergone comprehensive physical examination as a part of opportunistic screening, which were very difficult to include these subjects in other health examination institutions, especially patients with benign nodules. In the control group, the proportion of pulmonary nodules that disappeared in the follow‐up were diagnosed as benign nodules was relatively high. The above reasons may lead to different results of internal test and external verification.

In recent years, with the popularization of data mining technology and the development of deep learning methods such as convolutional neural network, the ability to predict the risk of lung cancer in patients using clinical data has become more frequent. Many studies focus on the prediction of treatment response and prognosis in patients with confirmed lung cancer.^39^, ^40^, ^41^, ^42^, ^43^ Some studies have predicted the risk of lung cancer through well‐known clinical indicators such as age, smoking history, past tumor history, asbestos exposure, COPD, weight, physical activity, and fasting blood glucose level.^44^ Based on the deep learning of image data,^45^, ^46^, ^47^, ^48^, ^49^ or with the help of a tracer,^50^, ^51^, ^52^, ^53^, ^54^, ^55^, ^56^ it has become easier to determine whether pulmonary nodules are benign or malignant. Furthermore, some researchers have developed computer‐aided targeting systems using these technologies.^57^ Some studies have also used clinical indicators to determine the risk of benign and malignant pulmonary nodules in patients.^58^, ^59^ However, a major limitation to these previous studies is that they have relied on a few indicators which have only been loosely established as having an association with lung cancer risk. Since the body is a highly complex and interconnected system, a clear cause–effect relationship between these commonly used indicators and lung cancer cannot be established and changes in some of these indicators are easy to ignore. The novelty and importance of the present study is that we have implemented machine learning to generate a risk model for lung cancer using data that is routinely captured in most physical examinations. This model provides clinicians with a valuable tool to assist in the diagnosis of benign and malignant pulmonary nodules.

### 4.1 Conclusion

For the subjects with negative or clinically tentative benign nodules in the initial LDCT screening, multiple LDCT re‐examinations within 5 years of follow‐up did not seem to have any further clinical value than the initial LDCT screening. Further analyses using data mining and machine learning technology found that for the population with pulmonary nodules identified by LDCT, it was feasible to use 15 variables routinely captured in physical examination to establish a risk model for predicting whether the pulmonary nodules were benign or malignant. The model was determined to be concise and effective and was validated using an external data set, which shows the model has generalizability and could be widely implemented.

A limitation of this study includes the small sample size of the early lung cancer and benign nodule groups. Additionally, the benign nodules detected with LDCT in this study were those assumed to not change during the follow‐up of at least 5 years, but there was no pathological evidence to confirm their benign status. Moreover, subjects used in this study were from the population of patients who have undergone comprehensive physical examination as a part of opportunistic screening. As such, this population may not fully represent ordinary, otherwise healthy individuals. Finally, the sample size of the cohort used for external validation was small. Future studies should validate this model in a much larger patient population to further test the generalizability and feasibility of its use as a clinical tool for predicting lung cancer risk.

## AUTHOR CONTRIBUTIONS

All authors meet the criteria for authorship stated in the Uniform Requirements for Manuscripts submitted to Biomedical Journals. Qiang Zeng and Miao Liu designed this study. Yan‐song Zheng, Jing Dong, and Xue Yang wrote the original drafts. Ping Shuai and Yongli Li acquired and analyzed the data. Hailin Li was responsible for methodology. Sheng‐yong Dong and Yan Gong reviewed and edited the manuscript. Yan‐song Zheng, Jing Dong, and Xue Yang contributed equally as co‐first authors. All authors read and approved the final manuscript. We agree to the terms of the BioMed Central Copyright and License Agreement.

## FUNDING INFORMATION

This study has received funding by the National Key R&D Program of China (2017YFC1308700). This was not an industry‐backed study.

## CONFLICT OF INTEREST STATEMENT

None of the authors have any affiliation or financial involvement that conflict with the material presented in this study.

## ETHICAL APPROVAL

The study protocol was approved (S2021‐427‐01) by the Chinese People's Liberation Army General Hospital ethics committee and complied with the principles of the Declaration of Helsinki and its contemporary amendments.

## CONSENT FOR PUBLICATION

Not applicable.

## Data Availability

Study data are available from the corresponding author upon request.
